# The Treatment of Inebriety by Atropine and the So-Called "Gold Cure."

**Published:** 1904-05-14

**Authors:** 


### THE TREATMENT OF INEBRIETY BY ATROPINE AND THE SO-CALLED "GOLD CURE."

Short-term systems of treating alcoholic excess are discussed in two papers contributed to the *British Medical Journal*. Dr. McBride, of Toronto, has had considerable experience of hypodermic injection of atropine and strychnine in cases of inebriety. His method is to give a hypodermic injection of atropine sulphate thrice daily, commencing with $\frac{1}{200}$ of a grain, and increasing the dose gradually until the pupils are affected; this maximum dose is maintained for about 14 days and then stopped or gradually reduced. At the same time injections of strychnine nitrate are used, commencing with $\frac{1}{60}$ grain and rising to $\frac{1}{20}$ grain three or more times a day. This maximum dose of strychnine is continued some days after the atropine has been omitted. A mixture containing an ordinary dose of Extr. Cinchonæ liq. is ordered every three hours. Diet and exercise are also regulated and the general health attended to.

Dr. McBride describes four cases in which this treatment succeeded. In a case of periodic alcoholism the treatment lasted six weeks. The patient lost his craving for stimulants on the third day, his insomnia disappeared on the fourth, and he has continued without a relapse for six years. The second patient, a man aged 45, had been drinking to excess for five years; he was markedly anæmic and his nervous system was shattered. On the second day he developed delirium tremens, and the treatment had to be suspended. Subsequently he was treated for about six weeks, and the cure has lasted seven years. Another man, aged 32 years, who was anæmic and nervous, and had been drinking excessively for three years, was treated more or less against his will and he continued to dose himself with whisky at frequent intervals. On the third day he could not drink a glass of whisky and soda. The course lasted five weeks, and this patient has had a great aversion to alcohol for the last 13 years. The fourth case was that of a commercial traveller, aged 50, who had been drinking to excess for 15 years. He had been fat and of exceptional ability, but was becoming thin and a nervous wreck. His liver was large and tender. After two days' treatment he developed delirium tremens and large doses of morphine were administered with success. The atropine and strychnine injections were continued, and in five weeks he left the sanatorium in what he termed "the pink of condition." He has remained well for 12 years.

Dr. Backnell Fenn states that he has treated a large number of inebriates on the "gold cure" system with a fair measure of success. The cure consists of (1) attention to the general health; (2) hypodermic injections of daturine ($\frac{1}{200}$ gr.) or atropine sulphate ($\frac{1}{500}$ gr.) and strychnine sulphate ($\frac{1}{100}$ gr.); (3) a mixture containing chloride of gold and sodium gr. $\frac{1}{20}$, ammonium chloride gr. i., aloin gr. $\frac{1}{25}$, fluid extract of viburnum ♏ x., and tincture cinchonæ ♏ xl., given every two hours day and night; and (4) a hydrotherapeutic course, including various kinds of baths.

Dr. Fenn, recognising that alcoholism is not only a simple physical disease but also a moral malady, seeks to lead the patient to look on life in a new way, and hopes for much from moral and mental persuasion. He states that about 60 per cent. of inebriates treated in this way remain total abstainers after a period of from one to eight years.

